# *Koenigia bingchachaensis* (Polygonaceae), a Remarkable New Species from the Alpine Subnival of Bingchacha, Zayü, Xizang, China

**DOI:** 10.1002/ece3.73290

**Published:** 2026-03-28

**Authors:** Bo Xu, Hang Sun, Dong Luo

**Affiliations:** ^1^ College of Biological and Food Engineering Southwest Forestry University Kunming Yunnan China; ^2^ Yunnan Key Laboratory for Plant Diversity and Biogeography of East Asia Kunming Institute of Botany, Chinese Academy of Sciences Kunming Yunnan China; ^3^ State Key Laboratory of Plant Diversity and Specialty Crops Kunming Institute of Botany, Chinese Academy of Sciences Kunming Yunnan China

**Keywords:** alpine subnival, *Koenigia bingchachaensis*, new species, phylogenetics, Polygonaceae, Qinghai–Tibet plateau, Zayü

## Abstract

A new species, *Koenigia bingchachaensis* Bo Xu & H. Sun (Polygonaceae), is described and illustrated based on material from the alpine subnival of Zayü County, Xizang, China. The species is characterized by a perennial tufted habit, extensively developed braided‐fissured rhizomes, numerous simple spreading stems, prominently petiolate (1.5–4.0 cm) and arched leaves, and paniculate inflorescences borne both terminal and axillary with a short, twisted rachis. Morphologically, it is closely allied to *K. tortuosa* and *K. hookeri* , but can be readily distinguished from both by a stable combination of vegetative and reproductive characters. Molecular phylogenetic analyses based on complete plastome and nuclear ribosomal ITS sequences strongly support *K. bingchachaensis* as a distinct species, resolving it as a well‐supported sister lineage to the clade comprising *K. hookeri* and *K. tortuosa*. The integrative taxonomic approach, combining detailed morphology with molecular data, unequivocally confirms the specific status of *K. bingchachaensis*. The discovery expands the known diversity of *Koenigia* and highlights the potential for uncovering unique plant lineages in the extreme alpine environments of the Qinghai–Tibet Plateau.

## Graphical abstract

*Koenigia bingchachaensis* Bo Xu & H. Sun (Polygonaceae), a remarkable new species from the alpine subnival of Zayü County, Xizang, China, is described and illustrated. The integrative evidence from morphology and phylogenetics confirms the status of *K. bingchachaensis* as a new species, which also highlights the ongoing discovery of unique biodiversity in poorly explored alpine regions of the Qinghai–Tibet Plateau.

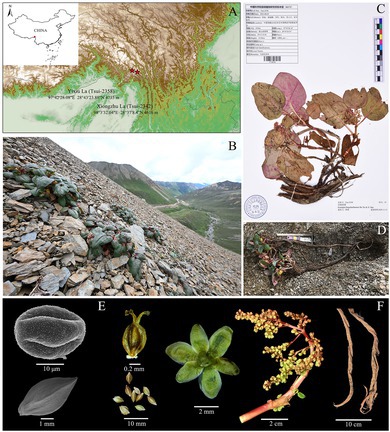

## 1 Introduction

*Koenigia* L. (Polygonaceae Juss.) is a morphologically diverse genus of arctic‐alpine herbs distributed across the Northern Hemisphere. It is currently estimated to comprise approximately 46 species according to Plants of the world Online (POWO). The diversity center of the genus lies in the Qinghai–Tibet Plateau and the adjacent Himalayan–Hengduan Mountains region (Hedberg 1997).

*Koenigia* was established by Linnaeus (1767) as a monotypic genus comprising only *K. islandica* Linnaeus (1767: 35), a delicate annual herb with markedly reduced floral structures. These include three tepals, three stamens alternating with the tepals, and a dimerous or trimerous gynoecium (Gross 1913; Galle 1977; Ronse Decraene 1989). These floral features depart strikingly from the typical polygonaceous flower, which has five tepals, eight stamens in two whorls, and a trimerous gynoecium. Consequently, *Koenigia* was treated separately from allied taxa for over a century (Ledebour 1850; Steward 1930; Roberty and Vautier 1964; Tutin et al. 1991; Li 1998; Li et al. 2003).

A broader concept of *Koenigia* was first proposed by Hedberg (1946) and later supported by morphological, anatomical, and palynological studies. These studies integrated species previously placed in traditional *Polygonum* sect. *Cephalophilon* and sect. *Aconogonon* (Marek 1954; Haraldson 1978; Ronse Decraene and Akeroyd 1988; Ronse Decraene et al. 2000). Hedberg (1997) formalized this expanded circumscription in a comprehensive revision of *Koenigia* s.l., primarily based on shared pollen characteristics and floral anatomy, recognizing six species and one subspecies.

Recent molecular phylogenetic studies have demonstrated that *Koenigia* belongs to tribe Persicarieae within subfamily Polygonoideae. The genus is closely related to *Aconogonon*, *Bistora*, and *Persicaria* (Sanchez et al. 2009, 2011). Fan et al. (2013) suggested to exclude *K. delicatula* from *Koenigia* s.s. due to its paraphyletic position at the base of the *Koenigia* + *Aconogonon* clade and axillary inflorescences, restricting the genus to five species. Subsequently, Schuster et al. (2015) proposed a broadened circumscription of *Koenigia* by transfering species traditionally assigned to *Aconogonon* into the genus. This resulted in a well‐supported monophyletic genus characterized by terminal inflorescences, distinctive pollen morphology, and specific floral and achene traits. Under this expanded concept, *Koenigia* comprises approximately 31 species. Subsequent regional taxonomic revisions have continued to refine species delimitation, leading to a current estimate of about 46 accepted species in the genus (POWO; Xue et al. 2024). Notably, recently described new species from the Eastern Himalayas (*K. chuanzangensis*, *K. arunachalensis*, and *K. medogensis* ) and central China (*K. hedbergii*) are included within this total, indicating that species diversity with the genus may still be underestimate (Min et al. 2015; Li et al. 2016; Hajong et al. 2025; Xie et al. 2025).

Despite recent advances in the taxonomy, *Koenigia* remains insufficiently studied, largely due to the absence of comprehensive taxonomic revisions based on extensive sampling across its distribution range. Many species are still poorly known, and additional undescribed diversity is likely to persist, particularly in floristically rich yet underexplored montane regions. This issue is especially pronounced in the alpine areas of the Eastern Himalayas, where complex topography, steep environmental gradients, and geographic isolation promote lineage diversification while constraining morphological divergence. Such conditions complicate species delimitation and may lead to an underestimation of species diversity.

**FIGURE 1:**
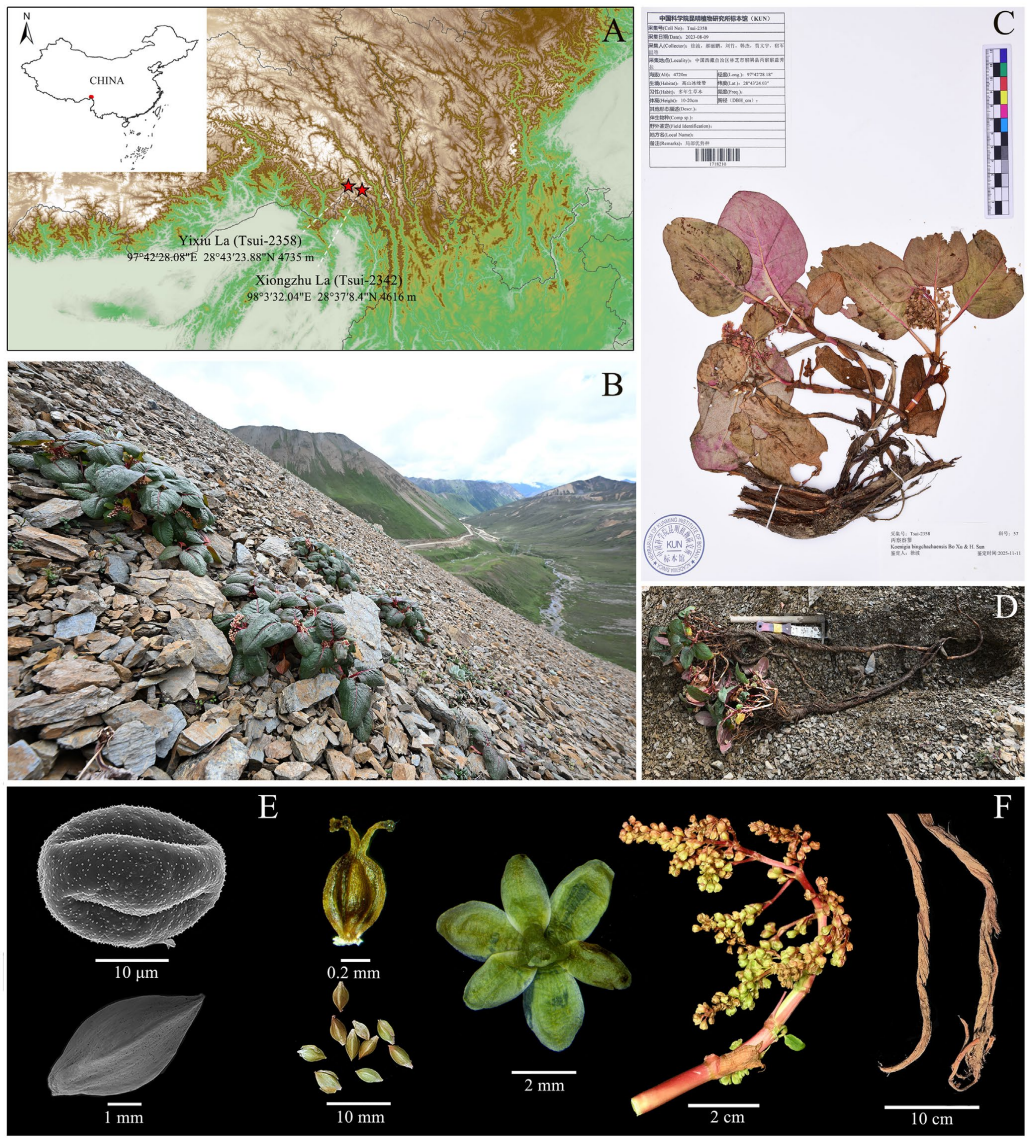
*Koenigia bingchachaensis*: (A) sampling location; (B) plant habit; (C) type specimen; (D) plant showing underground roots; (E) the scanning electron micrographs of pollen grains and achenes; and (F) dissected structures of roots, inflorescence, perianth, ovary, and seeds.

Nyingchi Prefecture, located in the Eastern Himalayas, exemplifies these conditions. The region is characterized by deeply incised alpine gorges and mountainous valleys, combined with a humid, oceanic monsoon climate. The interaction between complex topography and prevailing monsoonal airflow creates favorable hydrothermal conditions, making the region one of the biodiversity‐rich areas. During field surveys conducted from 2020 to 2025, we encountered two unusual populations of *Koenigia* on alpine subnival along the Bingchacha section of National Highway G219 in Zayü County, Nyingchi (Figure 1). These populations could not be confidently assigned to any previously described species, prompting further morphological and molecular investigation.

Detailed morphological comparisons, examination of herbarium specimens, and a comprehensive review of the relevant literature confirmed that these specimens represent a previously undescribed species. The new species is morphologically most similar to *K. tortuosa* and *K. hookeri* in its general inflorescence architecture. However, it can be consistently distinguished from both species by a stable combination of diagnostic characteristics. Specifically, *K. tortuosa* is a subshrub characterized by erect stems, dichotomous branching, and nearly sessile leaves. In contrast, *K. hookeri* is a herbaceous species with a single, unbranched upright stem, distinctly petiolate basal leaves (petioles 0.5–1 cm long), and unisexual, dioecious flowers. Based on this unique combination of morphological characters and supporting molecular phylogenetic evidence, we formally describe this taxon here as *Koenigia bingchachaensis* Bo Xu & H. Sun.

## 2 Materials and Methods

### 2.1 Field Works and Morphological Observations

Specimens were collected during fieldwork in August 2023 in Xiongzhu La and Yixiu La, in the Bingchacha section (the new Yunnan–Xizang corridor) of National Highway G219, Zayü, Xizang (Figure 1A). The type specimen (Tsui‐2358, Figure 1C) was deposited in the Herbarium of Kunming Institute of Botany (KUN). To assess morphological differences from related species, we examined specimens from Herbaria KUN, PE, K, A, E, and others, supplemented with online databases (JSTOR Global Plants, CVH, NSII, and GBIF).

Morphological descriptions were based on direct observations and measurements of living plants and herbarium specimens. Floral structures were dissected under a stereomicroscope (Sunyu/SOPTOP DS‐3000), and measurements were obtained using the Image View software. Mature pollen grains and achenes were collected for micromorphological observation (Zhou et al. 2004). Pollen grains were isolated from anthers, while achenes were fixed in FAA or rehydrated prior to dehydration through a graded ethanol series. Both pollen and achene samples were mounted on adhesive‐coated stubs; achenes were subsequently critical‐point dried. All samples were sputter‐coated with gold or platinum and examined using a field‐emission scanning electron microscope (Zeiss Sigma300) at an accelerating voltage of 10 kV. Multiple specimens were measured to assess intraspecific variation, and representative micrographs were captured to document diagnostic surface characters.

### 2.2 Phylogenetic Study

To assess the phylogenetic placement of *Koenigia bingchachaensis*, shallow whole‐genome sequencing (sWGS) was conducted on four individuals from Xiongzhu La and Yixiu La. Total genomic DNA was extracted and sequenced on an Illumina Novaseq 6000 platform. Complete plastomes and nuclear ribosomal internal transcribed spacer (ITS) regions were assembled from the sWGS data using GetOrganelle v1.7.4.1 (Jin et al. 2020) and annotated with PGA (Qu et al. 2019) using *K*. *ajanense* MZ573782 as the reference. Chloroplast genomes were visualized using Chloroplot (https://irscope.shinyapps.io/Chloroplot/).

Phylogenetic analyses were conducted separately for plastome and ITS datasets. Each dataset included four newly generated sequences of the putative new species, sequences from tribe Persicarieae (31 plastid, 26 cpDNA fragments, and 30 ITS sequences) and one outgroup from GenBank (Table S1), selected based on previous phylogenetic studies (Cao et al. 2022). Sequences were aligned using MAFFT v7.475 (Katoh and Standley 2013) and manually verified in Geneious v9.1.5 (https://www.geneious.com). Gaps were treated as missing data, and the best‐fit nucleotide substitution models were selected using the Akaike information criterion (AIC) in jModelTest (Darriba et al. 2012).

Phylogenetic relationships were inferred using Bayesian inference (BI) and maximum likelihood (ML) methods. BI analyses were performed in MrBayes v3.2.2 (Ronquist and Huelsenbeck 2003), with four Markov Chain Monte Carlo (MCMC) chains run for 2 million generations, sampling every 1000 generations. Convergence was assessed by the average standard deviation of split frequencies (< 0.01), and the first 25% of trees were discarded as burn‐in. ML analyses were conducted in RAxML v8.2.10 (Stamatakis 2014) under the GTR + GAMMA model with 1000 rapid bootstrap replicate. Phylogenetic trees were visualized in FigTree v1.3.1 (Rambaut 2010).

## 3 Results

Based on morphological examination, *Koenigia bingchachaensis* is a bisexual flower. The perianth is deeply divided into five or six segments; tepals are elliptic or obovate, 3–4 mm long and 1.5–3 mm wide, unequal in size, with 3–5 veins and a rounded apex. There are eight stamens, included; styles 3; stigmas capitate. The achene is enclosed within the persistent perianth, ellipsoid, trigonous, 3–4 mm long, constricted at the base into a short stipe, with an acute apex (Figure 1F). Scanning electron microscopy (SEM) observations show that the pollen grains of this species are elliptical and tricolpate, with a prominently spinulate exine. The distinctly echinate wall is a typical palynological characteristic of the genus *Koenigia* s.l. Additionally, SEM examination of the fruits reveal that they are distinctly trigonous and bear faint, irregular ornamentation (Figure 1E).

**FIGURE 2:**
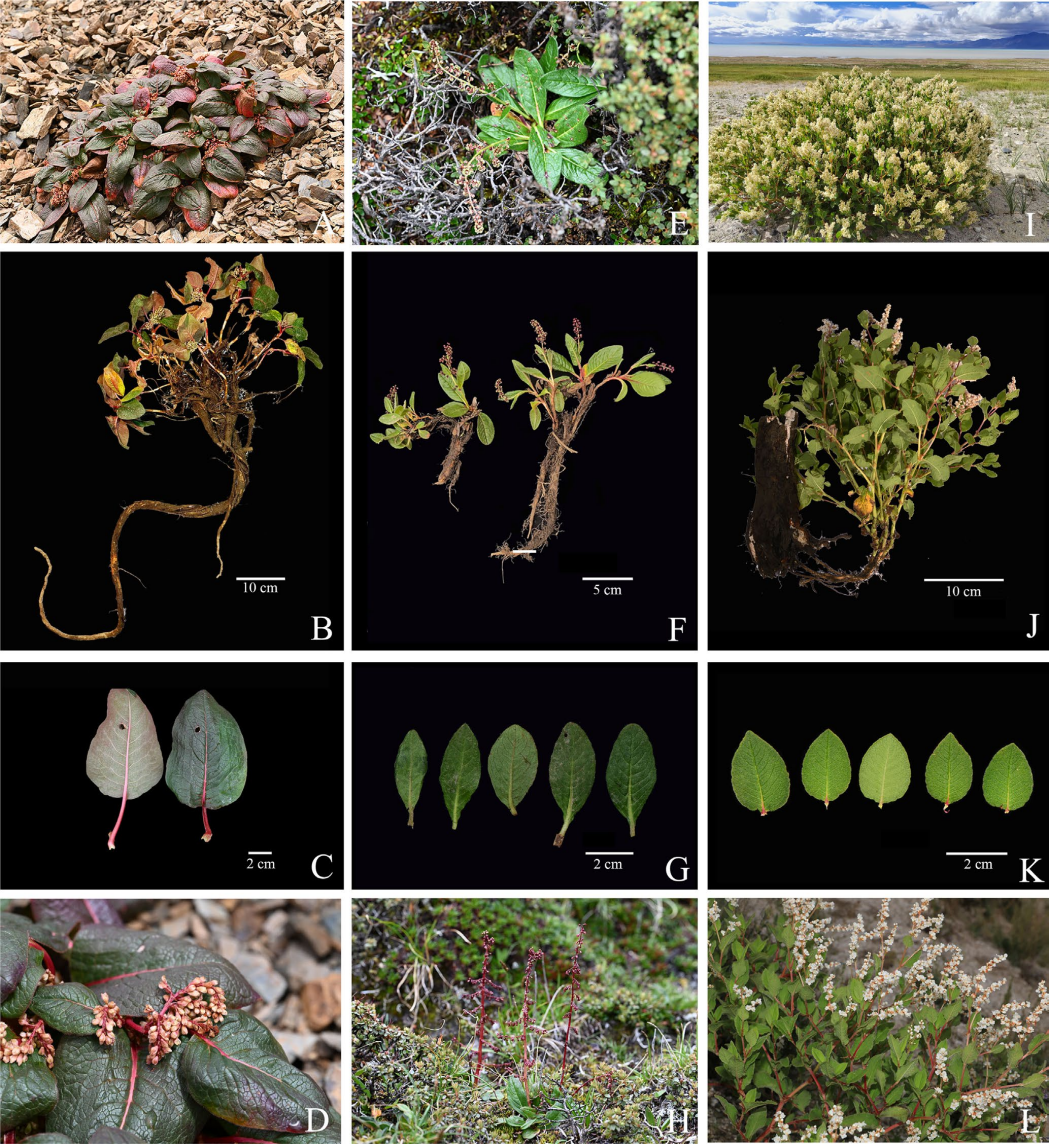
Morphological comparison among *Koenigia bingchachaensis* and its close relatives. (A–D) *K. bingchachaensis*; (E–H) *K. hookeri* ; (I–L) *K. tortuosa*. From top to bottom: Habitat, roots, leaves, and inflorescences.

Two species with terminal panicles that are morphologically similar to *Koenigia bingchachaensis*. *K. tortuosa* and *K. hookeri* were selected for morphological comparison because they share several general traits with *K. bingchachaensis* (Table 1; Figure 2). *K. tortuosa* is predominantly distributed in Xizang, often forming a dominant component of vegetation in high‐altitude arid regions at 3600–4900 m. *K. hookeri* occurs mainly in western Sichuan, northwestern Yunnan, eastern Xizang, southern Qinghai, and adjacent areas. It typically inhabits relatively humid alpine scrublands and meadows at 3500–5000 m. Multi‐year field investigations indicate that *K. bingchachaensis* is a narrow‐range endemic species, restricted to alpine subnival at elevations of 4600–4700 m. It represents typical alpine taxon adapted to extreme environments on the Qinghai–Tibet Plateau (Figure 1B). To date, only two populations have been discovered, both confined to the Bingchacha section of National Highway G219, Zayü County, Xizang (Figure 1A). Its distribution partially overlaps to some extent with that of *K. hookeri* .

**TABLE 1:** Morphological comparison among *Koenigia bingchachaensis*, *K. hookeri* , and *K. tortuosa*.

	*K. bingchachaensis*	*K. hookeri*	*K. tortuosa*
Life form	Perennial herb, tufts	Perennial herb	Perennial subshrub
Rhizome	Fleshy, elongated (to 100 cm), braided‐fissured	Fleshy, elongated (to 20 cm)	Lignified, elongated (to 60 cm)
Stem	Numerous, unbranched, spreading	Single, erect, unbranched	Erect, much‐branched
Leaves	Ovate to ovate‐cordate, 4–12 × 2–6 cm; petiolate	Narrowly elliptic or spatulate, 5–10 × 1.5–3 cm; shortly petiolate	Ovate to narrowly ovate, 1.5–4 × 1–2 cm; nearly sessile
Inflorescence	Paniculate, terminal, and axillary	Paniculate, terminal	Paniculate, terminal, and axillary
Flowers	Bisexual, yellowish‐green	Unisexual (dioecious); purple‐red	Bisexual; white
Achene	Ellipsoid, included	Broadly ovoid, slightly exerted	Ovoid, included

**FIGURE 3:**
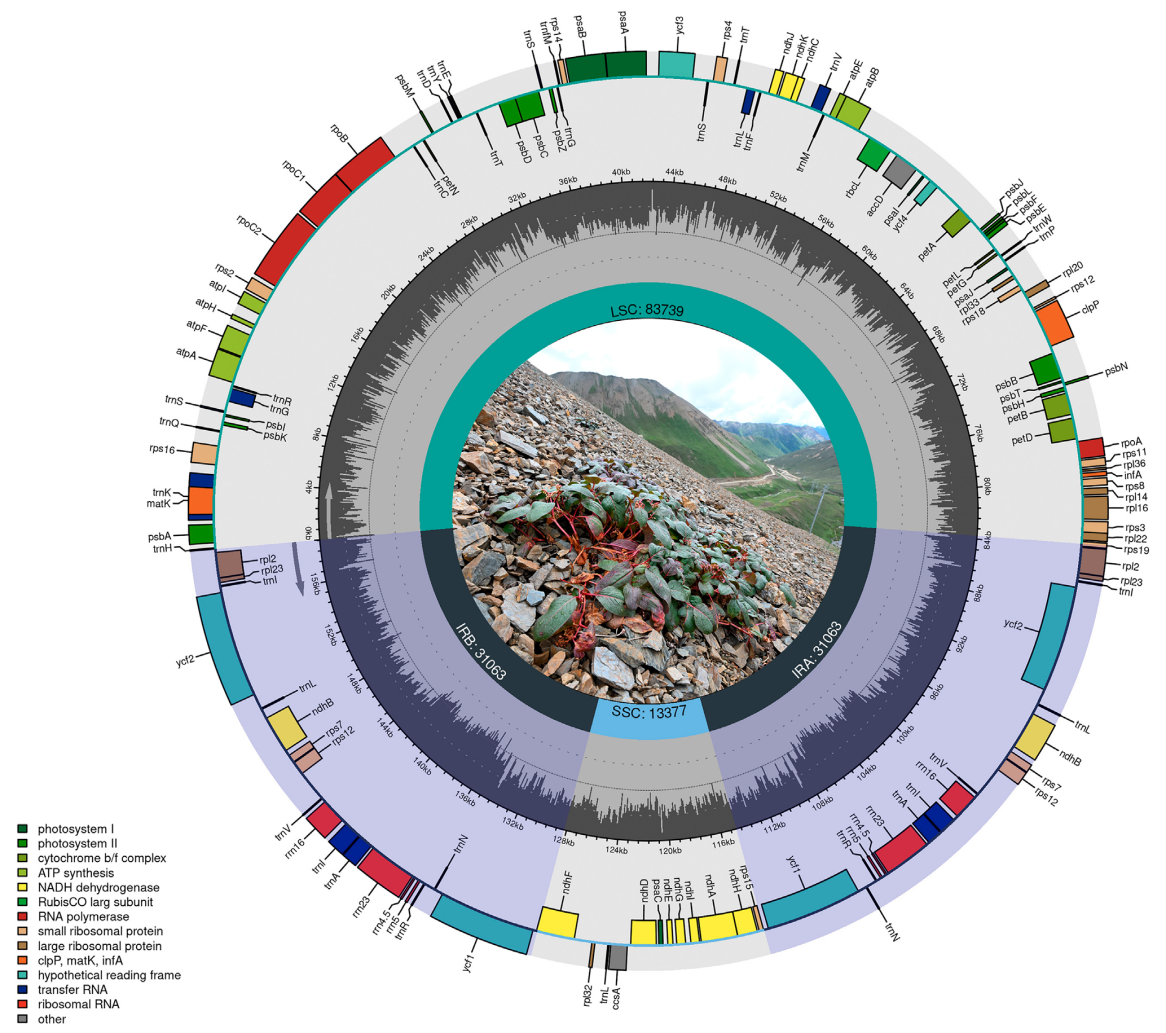
Plastome map of *Koenigia bingchachaensis*. Genes are color‐coded according to their functional categories. Abbreviations: IR, inverted repeat; LSC, large single‐copy; SSC, small single‐copy region.

**FIGURE 4:**
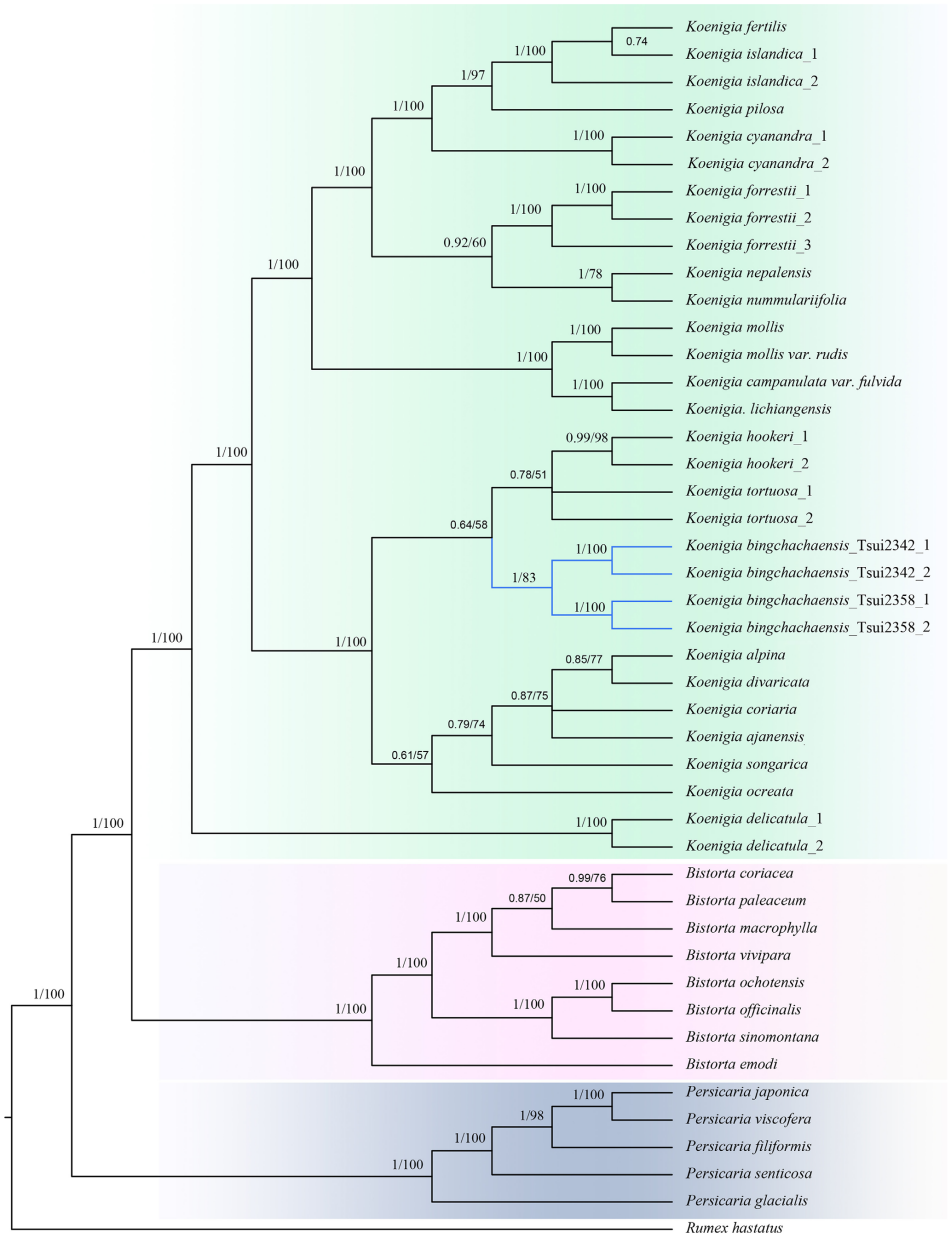
Phylogenetic relationships with tribe Persicarieae (Polygonaceae) inferred from complete chloroplast genomes using the Bayesian inference (BI) and maximum likelihood (ML) methods. Bayesian posterior probabilities (PP) and ML bootstrap (BS) values are presented above branches.

The plastomes of four *K. bingchachaensis* samples ranged from 159,242 to 159,248 bp (Figure 3) and exhibited the typical quadripartite structure, consisting of a large single‐copy (LSC), a small single‐copy (SSC), and two inverted repeat (IRa and IRb) regions. The ITS dataset comprises 35 sequences with an aligned length of 506 bp. Based on the Akaike information criterion (AIC), the best‐fit models for plastid and ITS datasets are TIM1 + I + G4 and GTR + G4, respectively. In the plastid phylogenetic trees, the four accessions of the putative new species form a well‐supported clade (PP = 1, BS = 100%; Figure 4), which is sister to the clade comprising *K. hookeri* and *K. tortuosa*. In the ITS trees, these four accessions also form a monophyletic lineage with moderate support (PP = 0.9, BS = 87%; Figure S1), and this lineage is sister to *K. hookeri* .

## 4 Discussion

The genus *Koenigia* exhibits substantial morphological variation. Under the narrow circumscription (*Koenigia* s.s.), it comprises delicate annual herbs with simplified floral structures—typically three tepals, three stamens, and a dimerous or trimerous gynoecium. The broader circumscription (*Koenigia* s.l.) incorporates these core species as well as members of Sect. *Aconogonon* (excluding *Knorringia sibirica*) and several species of Sect. *Cephalophilon* lacking capitate inflorescences (Hedberg 1946, 1997). Life forms range from delicate annuals (e.g., *K. islandica* ) to perennial herbs (e.g., *K. hookeri* ) and subshrubs (e.g., *K. tortuosa*); plant stature varies from creeping, slender stems to erect, robust species; leaves differ in size, shape, and texture; inflorescences vary from simple terminal clusters to large panicles; and floral anatomy ranges from three tepal flowers with two styles to five‐ or six tepal flowers with three styles. Additional distinguishing features include oblique and ruptured ochreas, deeply divided styles, and echinate pollen grains (POWO; Xue et al. 2024). The broad morphological spectrum has long complicated the classification of *Koenigia*. Although, some global databases (e.g., POWO) have adopted the broader circumscription, many Chinese floras and monograph ratain the narrower concept. The absence of a comprehensive systematic revision has caused nomenclatural confusion, with single species often corresponding to multiple names, posting challenges for nonspecialists.

Based on morphological characteristics and molecular phylogenetic studies (Cao et al. 2022), the putative new species was identified as a member of the genus *Koenigia*. Molecular phylogenetic tree from plastome and ITS datasets shows that the four accessions from two populations form a distinct clade. This clade is sister to *K. hookeri* (ITS) or to the clade of *K. tortuosa* and *K. hookeri* (plastid). Morphologically, the presence of terminal panicles indicates a close relationship with these species. While the combination of diagnostic traits—fleshy, elongated, braided‐fissured roots (Figure 1F); multiple unbranched stems arising from rhizome tips (Figure 1D), conspicuous petioles (1.5–4.0 cm); arched leaves densely covering the plant body; and both terminal and axillary paniculate inflorescences with short, twisted rachises (Figure 1F)—clearly distinguishes *K. bingchachaensis* from all other known *Koenigia* (Figure 1).

The alpine subnival belt, characterized by extreme elevation remoteness, has historically been poorly studied (Körner 2003; Sun 2023). *K. bingchachaensis* was first noticed in 2010 based on a photograph of an unusual polygonaceous plant (Dr. Ma Xiangguang, Sichuan University). The site's inaccessibility prevented further study until infrastructure improvements along the Bingchacha section of National Highway G219 allowed the collection of specimens and molecular materials starting in 2020. Field observations reveal that *K. bingchachaensis* exhibits clear adaptations to the alpine subnival zone (Körner 2003). Its fleshy, elongated roots, low‐growing and spreading stems, arching leaves that sheath the plant body, and concealed inflorescences likely mitigate the impacts of harsh winds and low temperatures in the alpine subnival zone. Despite well‐developed inflorescences, seed set is extremely low. Over several years of repeated field surveys, only a limited number of mature fruits were recorded, likely due to frequent rainfall during the flowering period, the structurally concealed flower that hinder pollination, and the scarcity of pollinating insects at high altitudes. Furthermore, the braided‐fissured rhizomes enable a single individual to produce multiple clonal ramets, partially compensating for low reproductive success. In autumn, as the aboveground parts wither and detach, the few mature fruits can be dispersed by wind along with plant debris, allowing the species to establish locally dominant vegetation patches.

Currently, only two stable populations are known, both restricted to the Bingchacha section of National Highway G219, Zayü County, Xizang, where their habitats are potentially threatened by road construction and occasional quarrying for gravel. Nevertheless, the species exhibits remarkable adaptation to extreme high‐altitude extreme environments, including clonal reproduction via braided‐fissured rhizomes and structural traits that mitigate environmental stress. These characteristics not only enable the species to persist under harsh conditions but also suggest its potential as a candidate for alpine vegetation restoration in degraded high‐elevation habitats.

## 5 Taxonomic Treatment

*Koenigia bingchachaensis* Bo Xu & H. Sun sp. nov. (Figure 1).

### 5.1 Type

CHINA. Xizang Autonomous Region: Nyingchi City, Zayü County, the Bingchacha section of National Highway G219, Yixiu_La Pass, 28°43′24.03″ N, 97°42′28.18″ E, elev. 4720 m, 9 Aug. 2023, Tsui‐2358 (holotype: KUN1718210!, Figure 1C; isotypes: CSH0221126!, HITBC0134683!, PE02717601!, SWFU‐Tsui‐2358!).

### 5.2 Diagnosis

Within the genus *Koenigia*, *K. bingchachaensis* is morphologically distinctive and highly diagnostic. Although it shares the characteristic of terminal paniculate with *K. tortuosa* and *K. hookeri* , the new species can be clearly differentiated by the following combination of features: its unique tufted growth habit; elongated and braided‐fissured rhizomes that may fragment; simple and spreading stems; and leaves with prominent petioles (1.5–4.0 cm long) that are arched and enclose the plant. Addtionally, its paniculate inflorescences occur both terminal and axillary, with a short, twisted rachis often concealed within the leaf cluster. Taken together, these morphological characteristics support its distinction from all other known species in the genus *Koenigia*.

### 5.3 Description

Perennial herbs. Rhizomes robust, ligneous, up to 100 cm long, fissured into a braided pattern. Stems numerous, tufted from rhizome, 10–20 cm tall, without basal leaves, simple, longitudinally ridged, red, spreading radially. Leaf blade ovate or ovate‐cordate, 4–12 cm long, 2–6 cm wide, arched and enclosing the plant; base broadly cuneate, rounded to slightly cordate, apex acute; margin entire, densely ciliate; adaxial surface glabrous, green; abaxial surface pubescent, yellowish‐brown to reddish‐brown; midvein pink to red. Petiole red, grooved, 1.5–4.0 cm long, glabrous to sparsely pubescent on the upper part. Ocrea tubular, membranous, ca. 2 cm long, oblique and lax at apex, glabrous. Inflorescence paniculate, terminal and axillary, sparsely pubescent. Rachis short, twisted, often concealed. Bracts brown, deltoid‐lanceolate, usually two‐flowered per bract. Pedicels glabrous, longer than bracts. Flowers bisexual. Perianth yellowish‐green to light green, transparent at margin, deeply 5 or 6‐parted; tepals elliptic or obovate, 3–4 mm long, 1.5–3 mm wide, unequally sized, 3–5‐veined, apex rounded. Stamens 8, included; anthers purple‐red. Styles 3; stigmas capitate. Achenes included in persistent perianth, yellowish‐green to brown, shiny, ellipsoid, trigonous, 3–4 mm long, constricted at base into a short stipe, apex acute.

### 5.4 Phenology

Flowering from July to August, fruiting from August to October.

### 5.5 Etymology

The specific epithet “bingchachaensis” refers to the renowned Bingchacha Highway (part of National Highway G219), also known as the new Yunnan‐Xizang corridor, which traverses a remote and rugged region of the Himalayas. The type specimen was collected along this very road. The construction of this highway has been pivotal in providing access to this previously inaccessible and botanically underexplored area, leading directly to the discovery of this new species. The name thus commemorates the highway's role as a unique link between human engineering and scientific discovery, a conduit that unveiled a hidden natural treasure.

### 5.6 Habitat and Distribution

Alpine subnival belt, including alpine scree slopes and gravel meadow, Zayü County, Nyingchi City, Xizang Autonomous Region, China. To date, this new species has only been discovered in two populations: Yixiu_La Pass and Xiongzhu_La Pass.

### 5.7 Additional Specimen Examined (Paratypes)

CHINA. Xizang. Nyingchi City, Zayü County, Yixiu_La Pass (25, Jun, 2020, Tsui‐1620 SWFU), and Xiongzhu_La Pass (8, Aug, 2023, Tsui‐2342 SWFU).

## 6 Conclusion

The discovery and formal description of *Koenigia bingchachaensis* is substantiated by robust evidence from comparative morphology and molecular phylogenetics. This species is distinguished by a unique combination of morphological traits—braided rhizomes, tufted growth habit, arching leaves, and concealed inflorescences—that represent remarkable adaptation to the harsh conditions of the alpine subnival belt. Phylogenetic analyses place it firmly within *Koenigia* s.l., as a sister lineage to the *K. hookeri‐K. tortuosa* clade, clarifying its systematic position and highlighting its evolutionary distinctiveness.

This finding advances our understanding of the biodiversity and speciation within *Koenigia*, a genus with a complex taxonomic history. It demonstrates the value of integrated approaches in resolving species boundaries in morphologically challenging groups. The restricted distribution and specific habitat of *K. bingchachaensis* further emphasizes the Eastern Himalayas' rugged, underexplored alpine region as hotspots of plant endemism. Moreover, improved accessibility provided by the Bingchacha Highway underscores how infrastructure can facilitate botanical discovery and conservation efforts. As a narrow‐range endemic potential vulnerable to habitat disturbance, this species merits targeted conservation attention. Future research should investigate population genetics, reproductive ecology, and comprehensive biogeographic patterns to fully unravel the evolutionary history of this intriguing alpine lineage.

## Author Contributions

**Bo Xu:** conceptualization (equal), data curation (lead), formal analysis (equal), funding acquisition (equal), writing – original draft (lead). **Hang Sun:** conceptualization (equal), funding acquisition (equal), project administration (equal), supervision (lead), writing – review and editing (equal). **Dong Luo:** conceptualization (equal), formal analysis (equal), supervision (equal), writing – review and editing (lead).

## Funding

This work was supported by the Second Tibetan Plateau Scientific Expedition and Research (STEP) program (2024QZKK0200) and Xingdian Talent Program to Bo XU (XDYCQNRC‐2022‐0215).

## Ethics Statement

The authors have nothing to report.

## Conflicts of Interest

The authors declare no conflicts of interest.

## Supporting information


**TABLE S1:** NCBI accession numbers of sequences used in phylogenetic analyses.
**FIGURE S1:** Phylogenetic relationships with tribe Persicarieae (Polygonaceae) inferred from ITS using the Bayesian inference (BI) and maximum likelihood (ML) methods. Bayesian posterior probabilities (PP) and ML bootstrap (BS) values are presented above branches.

## Data Availability

The datasets presented in this study can be found in online repositories below: http://ngdc.cncb.ac.cn/ under BioProject number PRJCA051437.

## References

[ece373290-bib-0001] Cao, D. L. , X. J. Zhang , X. J. Qu , et al. 2022. “Phylogenomics, Divergence Time Estimation, and Adaptive Evolution in the Polygonoideae (Polygonaceae).” Journal of Systematics and Evolution 61: 1004–1019. 10.1111/jse.12946.

[ece373290-bib-0002] Darriba, D. , G. L. Taboada , R. Doallo , and D. Posada . 2012. “jModelTest 2: More Models, New Heuristics and Parallel Computing.” Nature Methods 9: 772. 10.1038/nmeth.2109. PMC4594756 22847109

[ece373290-bib-0003] Fan, D. M. , J. H. Chen , Y. Meng , J. Wen , J. L. Huang , and Y. P. Yang . 2013. “Molecular Phylogeny of *Koenigia* L. (Polygonaceae: Persicarieae): Implications for Classification, Character Evolution and Biogeography.” Molecular Phylogenetics and Evolution 69: 1093–1100. 10.1016/j.ympev.2013.08.018. 23994356

[ece373290-bib-0004] Galle, P. 1977. “Untersuchungen zur Blutenentwicklung der Polygonaceen.” Botanische Jahrbücher für Systematik 98: 449–489. https://www.deutsche‐digitale‐bibliothek.de/item/RJZAQKRLKAJJFST2NROQHDKD3WULHWD7.

[ece373290-bib-0005] Gross, H. 1913. “Beitrage zur Kenntnis der Polygonaceae.” Botanische Jahrbiicher Fiir Systematik 49: 234–339.

[ece373290-bib-0006] Hajong, B. , M. Liden , and P. Bharali . 2025. “ *Koenigia arunachalensis* sp. Nov. (Polygonaceae), A New Species From Eastern Himalaya, India.” Nordic Journal of Botany 2025: e04750. 10.1002/njb.04750.

[ece373290-bib-0007] Haraldson, K. 1978. “Anatomy and Taxonomy in Polygonaceae Subfamily Polygonoideae Meisn. Emend. Jaretzky.” Symbolae Botanicae Upsalienses 22: 1–95. https://cir.nii.ac.jp/crid/1572543025405833344.

[ece373290-bib-0008] Hedberg, O. 1946. “Pollen Morphology in the Genus *Polygonum* L. s. l. and Its Taxonomical Significance.” Svensk Botanisk Tidskrift 40: 371–414. https://cir.nii.ac.jp/crid/1572824498944382208.

[ece373290-bib-0009] Hedberg, O. 1997. “The Genus *Koenigia* L. Emend. Hedberg (Polygonaceae).” Botanical Journal of the Linnean Society 124: 295–330. 10.1111/j.1095-8339.1997.tb01999.x.

[ece373290-bib-0010] Jin, J. J. , W. B. Yu , J. B. Yang , et al. 2020. “GetOrganelle: A Fast and Versatile Toolkit for Accurate de Novo Assembly of Organelle Genomes.” Genome Biology 21: 241. 10.1186/s13059-020-02154-5. 32912315 PMC7488116

[ece373290-bib-0011] Katoh, K. , and D. M. Standley . 2013. “MAFFT Multiple Sequence Alignment Software Version 7: Improvements in Performance and Usability.” Molecular Biology and Evolution 30: 772–780. 10.1093/molbev/mst010. 23329690 PMC3603318

[ece373290-bib-0012] Körner, C. 2003. Alpine Plant Life: Functional Plant Ecology of High Mountain Ecosystems. 2nd ed. Springer.

[ece373290-bib-0013] Ledebour, C. F. 1850. Flora Rossica. Vol. 3, 684. E. Schweizerbart.

[ece373290-bib-0014] Li, A. J. 1998. “Polygonaceae.” In Flora Reipublicae Popularis Sinicae, edited by A. J. Li , vol. 25, 1–237. Science Press.

[ece373290-bib-0015] Li, A. J. , B. J. Bao , A. E. Grabovskaya‐Borodina , et al. 2003. “Polygonaceae.” In Flora of China, edited by A. J. Li , vol. 5, 277–350. Science Press, Botanical Garden Press.

[ece373290-bib-0016] Li, B. , S. F. Chen , Y. Li , et al. 2016. “ *Koenigia hedbergii* (Polygonaceae: Persicarieae), A Distinct New Species From Shennongjia National Nature Reserve, Central China.” Phytotaxa 272: 116–122. 10.11646/phytotaxa.272.2.2.

[ece373290-bib-0017] Linnaeus, C. 1767. Mantissa Plantarum, 588. Laurentii Salvii.

[ece373290-bib-0018] Marek, S. 1954. “Morphological and Anatomical Features of the Fruits of Genera *Polygonum* L. and *Rumex* L. and Keys for Their Determination.” Monographiae Botanicae 2: 77–161.

[ece373290-bib-0019] Min, Y. J. , Z. Z. Zhou , X. X. Zhao , P. Gao , and C. Long . 2015. “ *Koenigia chuanzangensis* (Polygonaceae), A New Species From Western Sichuan and Eastern Xizang, China.” Novon: A Journal for Botanical Nomenclature 24: 266–272. 10.3417/2012071.

[ece373290-bib-0020] Qu, X. J. , M. J. Moore , D. Z. Li , and T. S. Yi . 2019. “PGA: A Software Package for Rapid, Accurate, and Flexible Batch Annotation of Plastomes.” Plant Methods 15: 50. 10.1186/s13007-019-0435-7. 31139240 PMC6528300

[ece373290-bib-0021] Rambaut, A. 2010. FigTree, version 1.3.1. Institute of Evolutionary Biology, University of Edinburgh.

[ece373290-bib-0022] Roberty, G. , and S. Vautier . 1964. “Les genres de Polygonacees.” Boissiera 10: 7–128.

[ece373290-bib-0023] Ronquist, F. , and J. P. Huelsenbeck . 2003. “MrBayes 3: Bayesian Phylogenetic Inference Under Mixed Models.” Bioinformatics 19: 1572–1574. 10.1093/bioinformatics/btg180. 12912839

[ece373290-bib-0024] Ronse Decraene, L. P. 1989. “The Flower of *Koenigia islandica* L. (Polygonaceae): An Interpretation.” Watsonia 17: 419–423. https://archive.bsbi.org.uk/Wats17p419.pdf.

[ece373290-bib-0025] Ronse Decraene, L. P. , and J. R. Akeroyd . 1988. “Generic Limits in *Polygonum* and Related Genera (Polygonaceae) on the Basis of Floral Characters.” Botanical Journal of the Linnean Society 98: 321–371. 10.1111/j.1095-8339.1988.tb01706.x.

[ece373290-bib-0026] Ronse Decraene, L. P. , S. P. Hong , and E. Smets . 2000. “Systematic Significance of Fruit Morphology and Anatomy in Tribes Persicarieae and Polygoneae (Polygonaceae).” Botanical Journal of the Linnean Society 134: 301–337. 10.1111/j.1095-8339.2000.tb02356.x.

[ece373290-bib-0027] Sanchez, A. , T. M. Schuster , J. M. Burke , and K. A. Kron . 2011. “Taxonomy of Polygonoideae (Polygonaceae): A New Tribal Classification.” Taxon 60: 151–160. 10.1002/tax.601013.

[ece373290-bib-0028] Sanchez, A. , T. M. Schuster , and K. A. Kron . 2009. “A Large‐Scale Phylogeny of Polygonaceae Based on Molecular Data.” International Journal of Plant Sciences 170: 1044–1055. 10.1086/605121.

[ece373290-bib-0029] Schuster, T. M. , J. L. Reveal , M. J. Bayly , and K. A. Kron . 2015. “An Updated Molecular Phylogeny of Polygonoideae (Polygonaceae): Relationships of *Oxygonum*, *Pteroxygonum*, and *Rumex*, and A New Circumscription of *Koenigia* .” Taxon 64: 1188–1208. 10.12705/646.5.

[ece373290-bib-0030] Stamatakis, A. 2014. “RAxML Version 8: A Tool for Phylogenetic Analysis and Post‐Analysis of Large Phylogenies.” Bioinformatics 30: 1312–1313. 10.1093/bioinformatics/btu033. 24451623 PMC3998144

[ece373290-bib-0031] Steward, A. N. 1930. “The Polygoneae of Eastern Asia.” Contributions From the Gray Herbarium of Harvard University 5: 1–129.

[ece373290-bib-0032] Sun, H. 2023. Flora of the Alpine Subnival Belt of the Qinghai‐Tibet Plateau. China Foresty Publishing House.

[ece373290-bib-0033] Tutin, T. G. , N. A. Burges , J. R. Edmondson , et al. 1991. “Polygonaceae Juss.” In Flora Europaea, edited by T. G. Tutin , vol. 1, 91–108. Cambridge University Press.

[ece373290-bib-0034] Xie, X. T. , Y. M. Wei , Y. J. Chen , et al. 2025. “ *Koenigia medogensis* (Polygonaceae: Persicarieae), A Distinct New Species From Xizang, Southwestern China.” Ecology and Evolution 15: e72089. 10.1002/ece3.72089. 40900720 PMC12399572

[ece373290-bib-0035] Xue, J. H. , V. V. Chepinoga , and K. P. Ma . 2024. Checklist of Vascular Plants of North Asia. EDP Sciences.

[ece373290-bib-0036] Zhou, Z. Z. , X. P. Zhang , and R. X. Xu . 2004. “Pollen Morphology of *Koenigia* From China.” Acta Phytotaxonomica Sinica 42: 513–523.

