# Supplementary material for: Koenigia bingchachaensis (Polygonaceae), a Remarkable New Species from the Alpine Subnival of Bingchacha, Zayü, Xizang, China
Source: Ecol Evol. 2026 Mar 28;16(4):e73290. doi: 10.1002/ece3.73290 (PMC13107280; doi:10.1002/ece3.73290)
Supplement: Supplementary file 1 — TABLE S1: NCBI accession numbers of sequences used in phylogenetic analyses. FIGURE S1: Phylogenetic relationships with tribe Persicarieae (Polygonaceae) inferred from ITS using the Bayesian inference (BI) and maximum likelihood (ML) methods. Bayesian posterior probabilities (PP) and ML bootstrap (BS) values are presented above branches. [file ECE3-16-e73290-s001.docx]

**TABLE S1** NCBI accession numbers of sequences used in phylogenetic analyses.

| **Taxon** | **plastome** | **cpDNA region** | **ITS** |
| --- | --- | --- | --- |
| *Bistorta coriacea* | MW770449 |  |  |
| *Bistorta emodi* | MZ573781 |  |  |
| *Bistorta macrophylla* | ON229545 |  |  |
| *Bistorta ochotensis* | ON229547 |  |  |
| *Bistorta officinalis* | ON229548 |  | EU591968 |
| *Bistorta paleaceum* | ON229603 |  |  |
| *Bistorta sinomontana* | ON229549 |  |  |
| *Bistorta vivipara* | ON229551 |  |  |
| *Koenigia* [*ajanensis*](https://www.ipni.org/n/77353641-1) | MZ573782 |  |  |
| *Koenigia alpina* | MZ573783 |  |  |
| *Koenigia campanulata* |  |  | JN235111 |
| *Koenigia campanulata* var. *fulvida* | MZ573784 |  | HQ435342 |
| *Koenigia cathayana* |  |  | JN235109 |
| *Koenigia coriaria* |  | EU024774 (trnL-trnF) |  |
| *Koenigia cyanandra_*1 | MZ573785 |  | HQ435339 |
| *Koenigia cyanandra_*2 | MZ573786 |  |  |
| *Koenigia delicatula_*1 | MZ573787 |  | JN235112 |
| *Koenigia delicatula_*2 | MZ573788 |  | JQ360837 |
| *Koenigia divaricata* | MZ573789 |  |  |
| *Koenigia fertilis* |  | JN234955 (rbcL)  JN235036 (trnL-trnF)  JN234914 (atpB-rbcL) | JN235101 |
| *Koenigia filicaulis_1* |  |  | JF977848 |
| *Koenigia filicaulis_2* |  |  | GU444019 |
| *Koenigia forrestii_1* | MZ573790 |  | JN235107 |
| *Koenigia forrestii_2* | OM735566 |  | DQ406633 |
| *Koenigia forrestii_3* | OK661157 |  |  |
| *Koenigia hookeri*_1 |  | JN234960 (rbcL)  JN235041 (trnL-trnF)  JN235000 (rpl32) | JN187112 |
|  |  |  |  |
| *Koenigia hookeri_*2 |  | EU840457 (trnK)  EU840331 (psaA)  EU840373 (ndhF)  EU840289 (rbcL)  EU840415 (accD)  EU840541 (trnL-trnF) | JN235110 |
| *Koenigia islandica* | MZ573791 |  | JN235102 |
| *Koenigia islandica* | OK661154 |  | JN235103 |
| *Koenigia jurii* |  |  | GQ339915 |
| *Koenigia lichiangensis* | MZ573792 |  | HQ843144 |
| *Koenigia mollis* | MZ573793 |  | EF653687 |
| *Koenigia mollis* var. *rudis* | MZ573794 |  |  |
| *Koenigia nepalensis* | MZ573795 |  | JN235106 |
| *Koenigia nummulariifolia* | OM674663 |  | JN235105 |
| *Koenigia ocreata* |  | GQ244535 (trnL-trnF) |  |
| *Koenigia panjutinii* |  |  | GQ339916 |
| *Koenigia phytolaccifolia* |  |  | MF964039 |
| *Koenigia pilosa* |  | JN234967 (rbcL)  JN235048 (trnL-trnF)  JN234925 (atpB-rbcL)  JN235007 (rpl32-trnL) | JN235104 |
| *Koenigia polystachya_*1 |  |  | EU718497 |
| *Koenigia polystachya_*2 |  |  | JF977867 |
| *Koenigia polystachya_*3 |  |  | MF785502 |
| *Koenigia relicta* |  |  | GQ339917 |
| *Koenigia songarica_1* |  | EU024773 (matK) | JF922102 |
| *Koenigia songarica_*2 |  |  | MT923434 |
| *Koenigia tortuosa_*1 |  | JN234957 (rbcL)  JN235038 (trnL-trnF)  JN234916 (atpB-rbcL)  JN234997 (rpl32) | JN235108 |
| *Koenigia tortuosa_*2 |  | MF786792 (matK)  MF785777 (psaA)  MF786481 (rbcL) | HQ435341 |
| *Persicaria filiformis* | ON229571 |  |  |
| *Persicaria glacialis* | ON229574 |  |  |
| *Persicaria japonica* | ON229577 |  |  |
| *Persicaria senticosa* | ON229593 |  |  |
| *Persicaria viscosa* | ON229596 |  |  |
| *Rumex hastatus* | NC050928 |  |  |

**FIGURE S1** Phylogenetic relationships with tribe Persicarieae (Polygonaceae) inferred from ITS using the Bayesian inference (BI) and maximum likelihood (ML) methods. Bayesian posterior probabilities (PP) and ML bootstrap (BS) values are presented above branches.

**
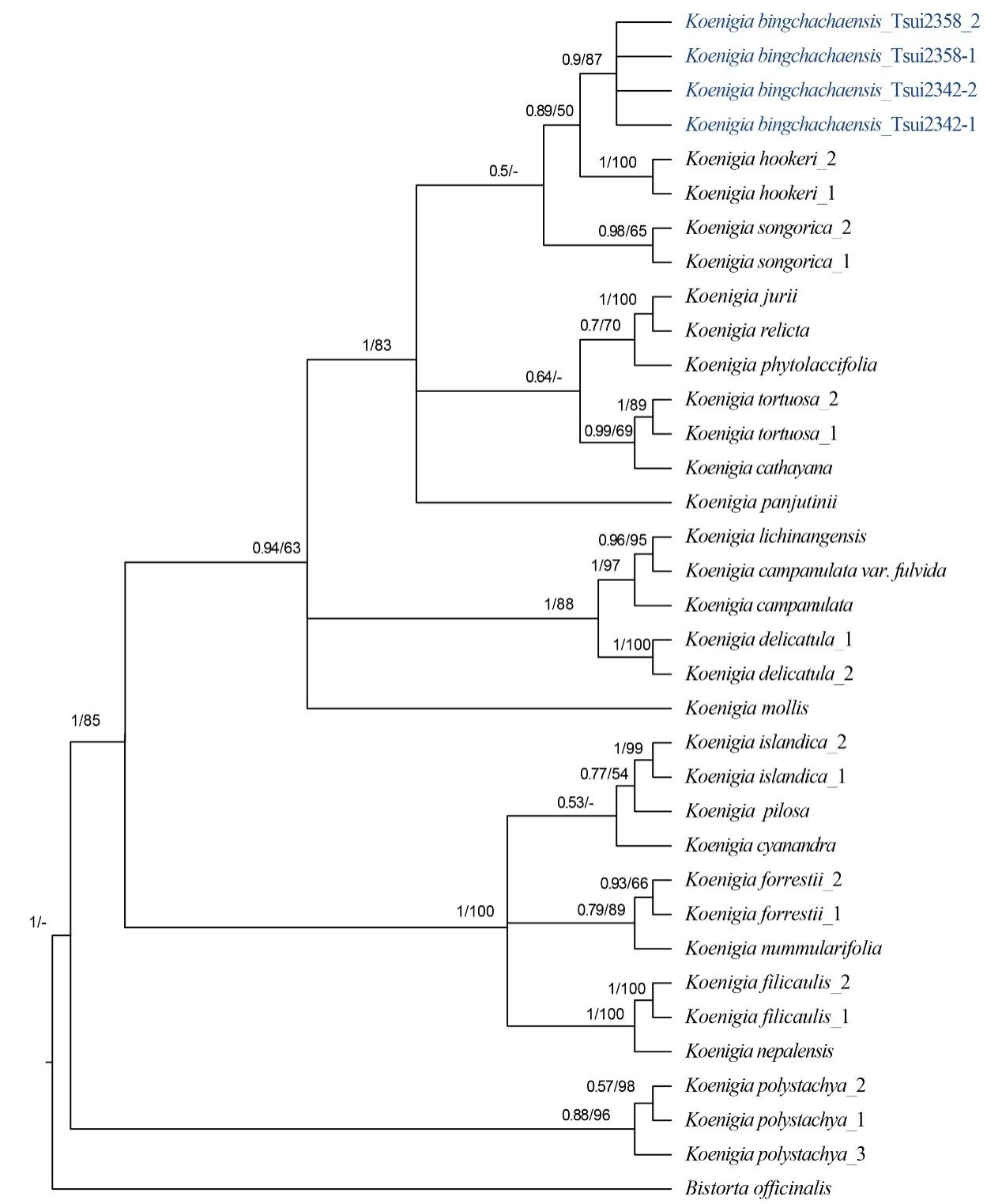
**
